# Molecular Regulation of Cardiomyocyte Cell Cycle and Regeneration

**DOI:** 10.3390/biology15070568

**Published:** 2026-04-02

**Authors:** Fan Li, Yura Son, Ming Yang, Wuqiang Zhu

**Affiliations:** 1 Department of Cardiovascular Medicine, Physiology and Biomedical Engineering, Center for Regenerative Biotherapeutics, Mayo Clinic Arizona, Scottsdale, AZ 85259, USA; son.yura@mayo.edu; 2 Department of Radiology, Mayo Clinic Arizona, Scottsdale, AZ 85259, USA; yang.ming@mayo.edu

**Keywords:** cardiomyocyte, cell cycle, proliferation, heart, regeneration

## Abstract

The regulation of the cardiomyocyte cell cycle is central to understanding heart regeneration. In adult mammals, cardiomyocytes withdraw from the cell cycle, limiting their ability to proliferate and repair heart tissue after injury. This process is controlled by intrinsic genetic programs and extrinsic signals, which together restrict the regenerative response in mature hearts. Understanding the mechanisms that regulate cardiomyocyte cell cycle activity is therefore critical for advancing cardiac regenerative medicine. In this review, we provide a comparative and integrated overview of cardiomyocyte cell cycle regulation in lower vertebrates and mammals, and discuss the major intrinsic factors that govern this process, including cyclin/CDK pathways, transcription factors and co-activators, oxygen and metabolic regulation, and epigenetic mechanisms. We also review the influence of hormones and growth factors, as well as the supportive roles of nonmyocyte populations in heart regeneration. By integrating findings across species, developmental stages, and regulatory levels, these findings highlight the complex regulatory network controlling cardiomyocyte proliferation and provide insight into potential therapeutic strategies for stimulating cardiac repair in the adult mammalian heart.

## Simple Summary

The adult mammalian heart has very little ability to regenerate after injury. Instead of replacing lost heart muscle, it usually forms scar tissue, which can weaken heart function and eventually lead to heart failure. In contrast, lower vertebrates and neonatal mammals retain a much stronger regenerative capacity. One major reason for this difference is that adult cardiomyocytes largely lose the ability to divide. Understanding how cardiomyocyte proliferation is controlled may help researchers develop new ways to repair the injured heart. In this review, we provide an integrated overview of cardiomyocyte cell cycle regulation by comparing evidence from lower vertebrates, neonatal and adult mammals, and multiple experimental models. We summarize the major molecular signals and cell types that regulate cardiomyocyte cell cycle activity and heart regeneration, and discuss how these findings may guide future regenerative therapies.

## 1. Introduction

Heart failure resulting from myocardial injuries remains a leading cause of morbidity and mortality worldwide [1]. In adult mammals, cardiomyocytes largely exit the cell cycle and have very limited capacity to re-enter proliferation in response to injury. As a result, injured myocardium is replaced with fibrotic scar tissue and undergoes pathological hypertrophy, both of which contribute to compromised cardiac function and altered electrophysiology [2]. The identification of critical molecular factors and signaling pathways involved in cardiomyocyte proliferation and tissue repair has generated new opportunities for developing therapeutic strategies to enhance cardiac regeneration and reduce fibrosis in the adult mammalian heart. Compared with previous reviews, the present review provides a comparative, cell cycle-centered synthesis of cardiomyocyte proliferation across lower vertebrates and mammals. It integrates intrinsic regulators, extrinsic signaling pathways, and nonmyocyte contributions to provide a broader framework for understanding heart regeneration.

In this review, we use operational definitions to standardize terminology. We define “cell cycle” as a general term referring to cardiomyocytes showing evidence of cell cycle activity; “cell cycle re-entry” denotes cardiomyocytes re-entering the cycle, as indicated by EdU/BrdU incorporation or Ki67 positivity; “mitosis” describes progression into M phase, exemplified by PH3 positivity; and “cytokinesis” refers to the completion of cell division, such as Aurora B localization at the midbody or cleavage furrow. Furthermore, we employ “cardiomyocyte proliferation” primarily to describe studies reporting increased cell cycle, mitotic, or division-related markers including pH3, Aurora B, or elevated cardiomyocyte numbers.

## 2. Cardiomyocyte Cell Cycle in Lower Vertebrates

Many lower vertebrates possess a robust capacity for cardiac regeneration. Amphibians such as newts and salamanders can fully restore heart structure and function after injury through cardiomyocyte dedifferentiation and proliferation. In adult eastern newts (*Notophthalmus viridescens*), this process results in complete recovery within approximately 70 days after damage [3,4,5,6]. Zebrafish also exhibit strong regenerative responses, regenerating nearly 20% of ventricular mass after apex amputation primarily through proliferation of GATA binding protein 4 (GATA4)-expressing cardiomyocytes [7,8]. To elucidate the origins of regenerated cardiomyocytes, transgenic zebrafish expressing tamoxifen-inducible Cre recombinase under the cardiomyocyte-specific cardiac myosin light chain 2 promoter were used for lineage tracing. These studies demonstrated that cardiac regeneration is driven predominantly by the proliferation of pre-existing cardiomyocytes rather than progenitor cells [9,10]. Furthermore, regeneration occurs predominantly through the proliferation of pre-existing cardiomyocytes, particularly those located in the injury border zone, which re-enter the cell cycle to initiate the regenerative process [8,10,11].

Proliferating cardiomyocytes dedifferentiate and adopt features resembling immature cardiomyocytes. Dedifferentiation is a phenomenon prominently observed during heart regeneration in zebrafish [7,10,12]. Cardiomyocytes dedifferentiation is characterized by sarcomeric disassembly with sarcomere displaced toward the cell periphery, partial cellular uncoupling, and induction of cell cycle-associated factors, enabling their re-entry into the cell cycle and contribution to myocardial repair [8,10]. Advanced sequencing techniques have provided further insight into the molecular changes underlying this regenerative response. RNA tomography and single cell RNA sequencing studies show the re-expression of embryonic cardiac genes, sarcomeric disassembly, suppression of mitochondrial gene expression, and a shift toward glycolytic metabolism in border zone cardiomyocytes [13,14]. Following injury, cardiomyocytes in the regenerating myocardium exhibit sarcomeric disorganization and impaired electrical conduction, which gradually recover over time [8,10]. Optical mapping studies by Kikuchi et al. demonstrated delayed conduction and reduced depolarization early after injury, followed by progressive restoration of electrical coupling and conduction velocity, with full functional integration achieved by approximately 30 days post-injury [8].

## 3. Cardiomyocyte Cell Cycle in Mammals

Mammalian hearts exhibit a transient regenerative capacity during early postnatal life that rapidly declines with maturation. In mice, robust cardiac regeneration is observed immediately after birth but is lost by postnatal day 7, after which injury leads to fibrotic scar formation [12]. Similar findings have been reported in rats, rabbits, sheep, and pigs, with fetal or early neonatal hearts displaying cardiomyocyte proliferation and functional recovery, whereas juvenile and adult hearts respond to injury with fibrosis and impaired function [15,16,17,18]. Transcriptomic analyses in large animals support these observations, showing heightened expression of cytokinesis-related genes in regenerating neonatal hearts and a shift toward fibrotic signatures with age [19]. Clinical observations in humans similarly suggest a brief neonatal window during which cardiac function can partially recover following injury associated with congenital heart disease [20,21]. Following this neonatal period, cardiomyocytes gradually exit the cell cycle as they undergo structural and functional maturation. Early studies and subsequent genetic analyses demonstrated that DNA synthesis and cardiomyocyte proliferation are extremely rare in adult mouse hearts, even after injury [22,23]. In humans, cardiomyocyte turnover decreases sharply after birth, eventually reaching a low steady-state rate of less than 1% per year by adolescence [24]. Postnatal cardiac growth is instead driven predominantly by cardiomyocyte hypertrophy rather than hyperplasia [25]. A notable feature of this proliferative-to-hypertrophic transition is polyploidization: in mice, most adult cardiomyocytes become binucleated through karyokinesis without cytokinesis, whereas in humans, cardiomyocytes largely remain mononuclear but acquire polyploid nuclei through DNA endoreplication [26,27,28,29,30].

The postnatal withdrawal of cardiomyocytes from the cell cycle is tightly coupled to progressive cardiomyocyte maturation. This maturation process involves several coordinated structural, electrophysiological, and metabolic remodelings that collectively reinforce permanent cell cycle exit [31]. Structurally, maturing cardiomyocytes undergo myofibril and sarcomere maturation, characterized by improved sarcomere alignment, increased sarcomere length, and isoform switching of contractile proteins from fetal to adult forms, including transitions in myosin heavy chains, myosin light chains, and troponin I isoforms [32,33,34,35]. Cardiomyocyte maturation is also accompanied by substantial electrophysiological remodeling, including hyperpolarization of the resting membrane potential, a longer plateau phase in the action potential, and reduced automaticity [31,36,37]. Cardiomyocytes in adult mammalian hearts form specialized cell–cell junctions called intercalated disks, maturing electrical and mechanical coupling between cardiomyocytes and further stabilizing the postmitotic state [31,38,39]. Finally, metabolic reprogramming from glycolysis toward mitochondrial oxidative phosphorylation, by the activation of metabolic transcriptional regulators such as peroxisome proliferator-activated receptor gamma coactivator 1 alpha/beta (PGC-1α/β), peroxisome proliferator-activated receptor alpha (PPARα), nuclear respiratory factor 1/2 (NRF1/2), and estrogen-related receptor alpha/beta/gamma (ERRα/β/γ), supports the energy demands of mature cardiomyocytes while coinciding with permanent cell cycle arrest [12,40,41].

## 4. Intrinsic Factors Regulating Cardiomyocyte Cell Cycle

The regulation of the cardiomyocyte cell cycle is orchestrated by a variety of molecular factors, transcription factors, and post-transcriptional regulators. While these pathways are highly active during embryonic development, they become progressively diminished after birth, enforcing the postmitotic state of adult cardiomyocytes.

### 4.1. The Cyclin and CDK Pathways

The cardiomyocyte cell cycle is controlled by cyclin-dependent kinase (CDK) complexes that regulate cell cycle progression (Table S1). In embryonic and neonatal hearts, cyclins and CDKs are expressed at high levels, supporting robust cell cycle activity; however, adult hearts exhibit downregulation of the positive cell cycle regulators and upregulation of cyclin-dependent kinase inhibitors (CKIs) [42]. Overexpression of cyclins such as cyclin A2 [43], cyclin D1 [44,45], and cyclin D2 [46] can stimulate the cardiomyocyte cell cycle. In particular, cyclin D2 overexpression not only activates the cell cycle in human-induced pluripotent stem cell-derived cardiomyocytes (hiPSC-CMs), but also improves engraftment after transplantation into infarcted mouse hearts [47,48,49]. Moreover, coordinated overexpression of CDK1 and CDK4 together with cyclin B1 and cyclin D1 has been shown to induce robust cell division in post-mitotic mice, rats, and hiPSC-CMs [50]. In contrast, triple knockdown of CKIs, including p21 (Waf1/Cip1), p27 (Kip1), and p57 (Kip2), induces re-entry of the cell cycle and promotes cytokinesis in both neonatal and adult cardiomyocytes [51]. Cyclin G1 provides additional regulatory complexity: its overexpression in neonatal cardiomyocytes increases DNA synthesis but inhibits cytokinesis, leading to multinucleation, whereas genetic deletion reduces polyploidization [52].

The p38 signaling pathway plays as a suppressor of cardiomyocyte cell cycle. Activation of p38 through MKK3bE (a constitutively active mutant of MAP kinase kinase 3b) decreases cardiomyocyte proliferation, whereas genetic deletion or pharmacological inhibition enhances cardiomyocyte proliferation [53]. Notably, p38 regulates the expression of key mitotic genes, including cyclin A and cyclin B [53]. Following cardiac injury in zebrafish, transient reduction in p53 activity promotes cardiomyocyte cell cycle during regeneration, while inhibition of its negative regulator murine double minute 2 (Mdm2) impairs regeneration [54]. In mice, loss of *p53* alone is insufficient to trigger DNA synthesis, whereas combined deletion of *p53* and *Mdm2* leads to a significant increase in cardiomyocyte proliferation in vivo, associated with decreased CKIs expression and increased cyclin E levels [55].

### 4.2. Transcription Factors/Co-Activators

Transcription factors integrate developmental cues with cell cycle control to determine cardiomyocyte proliferative capacity (Table S2). During embryogenesis and regeneration, pro-proliferative transcriptional programs promote cardiomyocyte expansion, whereas postnatal maturation is accompanied by the activation of transcriptional repressors that enforce cell cycle exit. Several developmental transcription factors directly regulate the cardiomyocyte cell cycle, including GATA4 [56], T-box transcription factor 20 (TBX20) [57,58,59,60], forkhead box K1 (FOXK1) and K2 (FOXK2) [61], E2F transcription factor 2 and 4 (E2F2 and E2F4) [62,63], as well as signaling pathways such as Notch [64,65,66], Wnt/β-catenin [67]. GATA4 directly induces the expression of cyclin D2 and CDK4, linking developmental signaling to canonical cell cycle machinery [56]. Similarly, FOXM1 are essential transcription factors for cardiomyocyte proliferation in regenerative models [68]. Cardiomyocyte-specific FOXM1 overexpression via modRNA promotes recovery from myocardial infarction (MI) in adult mice and pigs [69]. FOXK1/2 promote cardiomyocyte cell cycle progression by activating CCNB1/CDK1 and upregulating hypoxia-inducible factor 1 α (HIF1α) to enhance glycolysis and the pentose phosphate pathway [61]. In contrast, forkhead box O3 (FOXO3) and forkhead box P1 (FOXP1) act as brakes: *Foxo3* loss promotes postnatal cardiomyocyte proliferation and regeneration by relieving secreted frizzled related protein 2-mediated inhibition of Wnt/β-catenin signaling [70], while cardiomyocyte *Foxp1* loss enhances proliferation through a metabolic axis involving ubiquitin-specific peptidase 20, HIF1α, and heart and neural crest derivatives expressed 1 (Hand1), and AAV9-driven *Hand1* induction improves regeneration [71]. Reprogramming factors such as Octamer-binding transcription factor 4 (Oct4), SRY-box transcription factor 2 (Sox2), Krüppel-like factor 1 (KLF4) and c-Myc, have also been shown to induce dedifferentiation and proliferation in adult cardiomyocytes [72,73]. Notably, reduced expression of Krüppel-like factor 1 (KLF1) during postnatal development does not affect cardiomyocyte proliferation under basal conditions but is associated with a loss of myocardial regenerative capacity in neonatal injury models. Conversely, KLF1 overexpression promotes cardiomyocyte proliferation and enhances cardiac regeneration in adult mice after MI [74].

Wnt/β-catenin signaling is implicated in promoting cardiomyocyte proliferation and heart regeneration. Reactivation of Wnt/β-catenin signaling by increasing cytoplasmic β-catenin enhances BrdU incorporation in neonatal cardiomyocytes and proliferation in hESC-CMs [75]. Low-density lipoprotein receptor-related protein 6 (LRP6) is integral to the canonical Wnt/β-catenin pathway. *Lrp6* deficiency in mouse hearts increases cardiomyocyte proliferation and, through the inhibitor of growth family member 5/p21 pathway, reduces infarct size and improves cardiac function after MI [76]. More recently, N-cadherin is shown to promote the proliferation of neonatal mouse cardiomyocytes and hiPSC-CMs, whereas its deletion impairs neonatal regeneration and its overexpression enhances repair after adult ischemic injury. Mechanistically, N-cadherin stabilizes β-catenin and promotes its pro-mitotic transcriptional activity, thereby supporting cardiomyocyte self-renewal [77].

The Hippo pathway is a key regulator of cardiomyocyte proliferation and heart regeneration by restricting Yes-associated protein (YAP) activity [67]. Cardiomyocyte-specific knockout of Hippo signaling components, such as Salvador (*Sav1*), promotes myocardial regeneration and reduces scar size after cardiac injury [78]. Additional regulation occurs through ubiquitin-mediated control of MOB1B (MOB kinase activator 1B), a core coactivator that promotes large tumor suppressor kinase 1/2 activation and YAP/TAZ inhibition; cullin-associated and neddylation-dissociated 1 enhances F-box and WD repeat domain containing 11-dependent K48-linked ubiquitination and degradation of MOB1B, thereby relieving Hippo-mediated restraint on YAP signaling and promoting cardiomyocyte proliferation [79]. Conversely, activation of YAP1 stimulates postnatal cardiomyocyte proliferation and enhances cardiac regeneration and cardiac function following MI [80,81]. Consistent with this, expression of *YAP5SA*, a Hippo-resistant YAP variant, in adult mouse hearts drives cardiomyocytes to re-enter the cell cycle at the G1/S transition and progress into a prolonged S phase (~48 h); however, efficient progression into G2/M requires sarcomere disassembly and mitotic rounding, and overall mitosis/cytokinesis remains slow and is further constrained by p21 induction, indicating that YAP activation only partially overcomes adult cell cycle and DNA damage checkpoints [82]. Together, these transcriptional networks establish a balance between proliferative competence and permanent cell cycle arrest, coordinating cardiomyocyte differentiation, maturation, and regenerative potential.

Notably, Wnt/β-catenin and Hippo/YAP signaling are not independent pathways but intersect at multiple levels in a context-dependent manner. In cardiomyocytes, disruption of Hippo signaling (e.g., *Sav1* deletion) increases nuclear β-catenin, indicating derepressed canonical Wnt activity and supporting a model in which Hippo constrains heart growth in part by limiting Wnt/β-catenin signaling [78]. Conversely, cytoplasmic YAP/TAZ can suppress β-catenin-dependent transcription in certain contexts, and Wnt ligands can activate YAP/TAZ through β-catenin-independent mechanisms [83]. These observations justify the inclusion of both pathways as interconnected determinants of cardiomyocyte proliferative competence and regenerative potential.

In contrast, Meis homeobox 1 (MEIS1) acts as a key transcriptional brake on cardiomyocyte proliferation. Deletion of *Meis1* in mouse cardiomyocytes extends the postnatal proliferative window and promotes adult cardiomyocyte proliferation, whereas its overexpression suppresses regeneration by activating CKIs, including p15, p16, and p21 [84]. The MESI1 cofactor Homeobox B13 (HOXB13) similarly restricts proliferation, and combined knockout of *Meis1* and *Hoxb13* enhances proliferation and sarcomere disassembly and improves left ventricular systolic function following MI [85]. Additional repression pathways involve RB transcriptional corepressor 1 and MEIS2, further underscoring the importance of transcriptional control in maintaining the postmitotic state [86].

### 4.3. Oxygen and Metabolic Regulation

Oxygen availability and metabolic state are major extrinsic regulators of cardiomyocyte cell cycle activity (Table S3). An oxygen-rich postnatal environment induces cardiomyocyte cell cycle arrest through transition in mitochondrial metabolism and the impact of reactive oxygen species (ROS) on cardiomyocyte proliferation [87]. The increased mitochondrial ROS production activates the DNA damage response pathway, which in turn triggers cell cycle arrest in cardiomyocytes. Inhibition of ROS scavenging mechanisms or DNA damage response pathways delays cell cycle exit, underscoring their role in enforcing postnatal cell cycle arrest. Sirtuin 4 (*Sirt4*) overexpression in cardiomyocytes increases oxidative DNA damage and suppresses neonatal regeneration, whereas *Sirt4* deficiency promotes cardiomyocyte proliferation, extends the regenerative window, and improves adult repair after ischemia–reperfusion (I/R) injury in mice [88]. Conversely, systemic hypoxemia suppresses oxidative metabolism, reduces oxidative DNA damage, and promotes cardiomyocyte proliferation and myocardial regeneration, after MI [89]. Consistent with hypoxia-linked signaling being pro-regenerative, ectopic HIF2A enhances cardiomyocyte proliferation and improves cardiac function after injury in mice [90], and high mobility group box 2 (HMGB2) promotes regeneration in mice by activating a HMGB2/metastasis-associated 1 family member 2/HIF-1α axis that supports glycolytic programs [91].

Metabolic pathway rewiring further influences regenerative capacity, spanning glycolysis, fatty acid oxidation, and nucleotide and serine metabolism. Glycolysis is essential for cell cycle re-entry; inhibition of glycolysis impairs cardiomyocytes’ proliferative capacity, while enhancing mitochondrial oxidation by promoting pyruvate conversion to acetyl-CoA diminishes the number of proliferating cardiomyocytes following cardiac injury [92]. In addition, overexpression of pyruvate dehydrogenase kinase 3b, which favors the conversion of pyruvate to lactate, increases cardiomyocyte proliferation after injury [92]. Moreover, pharmacological inhibition of succinate dehydrogenase with malonate stimulates cardiomyocyte cell proliferation [93]. Lipid pathways regulate regeneration through fuel utilization as well as bioactive signaling. Suppressing fatty acid oxidation through acyl CoA synthase long chain family member 1 or carnitine palmitoyltransferase 1b deletion reverses cardiomyocyte cell cycle arrest [94,95]. In addition, overexpression of sphingosine kinase 2, an enzyme that produces sphingosine-1-phosphate, extends the postnatal regenerative window and improves adult repair after MI in mice, in part through nuclear inhibition of histone deacetylases [96]. Consistently, cardiomyocyte loss of sphingosine-1-phosphate receptor 1 (*S1pr1*) markedly reduces cardiomyocyte proliferation and impairs neonatal regeneration after apical resection, whereas AAV9-mediated cardiomyocyte-specific *S1pr1* gain-of-function enhances cardiac regeneration [97].

Along with this, purine synthesis is an additional metabolic requirement for the cardiomyocyte cell cycle. Deletion of the muscle-specific de novo purine synthesis enzyme adenylosuccinate synthase 1 (*Adssl1*) reduces neonatal cardiomyocyte proliferation and heart regeneration after apical resection, whereas cardiomyocyte-specific *Adssl1* overexpression extends the postnatal regenerative window and enhances cell cycle re-entry after MI, reducing scar size and improving cardiac function [98]. Purine metabolism can also be regulated at the level of nucleotide turnover. Targeting purine nucleotide degradation by inhibiting xanthine oxidase enhances cardiomyocyte proliferation and prolongs the postnatal regenerative window in neonatal mice [99]. Overexpression of phosphoglycerate dehydrogenase, the first and rate-limiting enzyme for serine biosynthesis from the glycolytic intermediate 3-phosphoglycerate, promotes myocardial regeneration and improves cardiac function after apical resection in neonatal mice and after MI in adult mice, whereas phosphoglycerate dehydrogenase inhibition blunts these effects [100]. Consistently, cardiac delivery of phosphoserine aminotransferase 1 (PSAT1) modRNA after MI promotes cardiomyocyte proliferation and repairs myocardium by activating the serine synthesis pathway to support nucleotide synthesis and reduce oxidative stress, and PSAT1 is required for YAP1-driven proliferation and enhances β-catenin nuclear translocation [101]. Additional hypoxia-linked regulation has been described through a metabolite-sensing transcriptional axis. C-terminal binding protein 2, which is activated during cardiomyocyte ischemia/hypoxia by intracellular NADH accumulation, promotes cardiomyocyte proliferation and heart regeneration via a FOXO1–p21/p27 pathway [102].

### 4.4. Epigenetic Regulation

Epigenetic regulation is an important mechanism controlling the cardiomyocyte cell cycle. Through DNA methylation, histone modifications, chromatin remodeling, and non-coding RNAs, epigenetic regulators influence the expression of genes involved in cell cycle progression, differentiation, and maturation. These mechanisms help enforce postnatal cardiomyocyte cell cycle arrest, whereas their modulation can promote cell cycle re-entry and regeneration after cardiac injury (Table S4).

#### 4.4.1. Chromatin-Based Epigenetic Regulation

DNA methylation, histone modifications, and chromatin remodeling are key determinants of cardiomyocyte cell cycle competence by reshaping chromatin states that control proliferative gene programs. In mouse cardiomyocytes, multiple studies converge on trimethylation of histone H3 lysine 27 (H3K27me3) as a major barrier to proliferation. Increasing α-ketoglutarate in mice extends the proliferative window and enhances regeneration after MI by reducing H3K27me3 at cell cycle gene promoters through jumonji domain-containing protein 3-dependent demethylation [95], and high mobility group A1 similarly promotes cardiomyocyte proliferation and regeneration in mice by lowering H3K27me3 levels [103]. Beyond methylation, chromatin remodeling and acetylation pathways also modulate regenerative potential. Prothymosin alpha (PTMA) promotes cardiomyocyte proliferation and regeneration across mouse and rat primary cardiomyocytes and human iPSC-CMs, and is required for neonatal heart regeneration in mice; mechanistically, PTMA inhibits deacetylation within the nucleosome remodeling and deacetylase complex containing methyl-CpG-binding domain protein 3 and histone deacetylase 1, thereby increasing the signal transducer and activator of transcription 3 (STAT3) acetylation and supporting STAT3 activation [104]. In addition, histone deacetylase 7 overexpression in neonatal mouse cardiomyocytes drives dedifferentiation-dependent proliferation through myocyte enhancer factor 2 suppression [105].

#### 4.4.2. Non-Coding RNAs

1. miRNAs are well-established post-transcriptional regulators that can promote cardiomyocyte cell cycle activity by targeting networks controlling DNA synthesis and cytokinesis. A genome-wide miRNA screen identified forty human miRNAs that significantly enhanced both DNA synthesis and cytokinesis in neonatal mouse and rat cardiomyocytes, with miR-590 and miR-199a emerging as potent inducers of cardiomyocyte proliferation [106]. These miRNAs promote cardiac regeneration, resulting in nearly complete restoration of cardiac function after MI. Additional miRNAs that enhance the cardiomyocyte cell cycle include miR-1248, miR-33b, miR-1825, miR-431, miR-130b-5p and members of the miR-17/92 and miR-302/367 clusters [107,108,109,110,111]. Notably, members of the miR-17/92 cluster, such as miR-19a and miR-19b, are upregulated in heart failure patients. Intracardiac injection of miR-19a/19b mimics improves cardiomyocyte proliferation and stimulates cardiac regeneration following MI [112]. Transient administration of miR-302/367 mimics has been shown to improve cardiac function after MI, whereas prolonged overexpression impairs cardiac function [108].

Mechanistically, many pro-proliferative miRNAs activate the YAP signaling. For example, miR-199a enhances YAP nuclear localization by directly targeting TAO kinase 1 (an upstream YAP inhibitory kinase) and beta-transducin repeat-containing protein (an E3 ubiquitin ligase responsible for YAP degradation) [113]. Several pro-proliferative miRNAs also modulate the depolymerization of actin through inhibition of Cofilin2, further promoting YAP nuclear translocation. Thus, activation of YAP and modulation of the actin cytoskeleton are central mediators of miRNA-induced cardiomyocyte proliferation [113]. While several miRNAs promote the cell cycle, others act as inhibitors of cardiomyocyte proliferation. miR-1 suppresses cyclin D1 expression, thus inhibiting the cardiomyocyte cell cycle in neonatal mouse cardiomyocytes [114]. Members of the miR-15 family, such as miR-195, restrict postnatal cardiomyocyte proliferation and impair neonatal heart regeneration, whereas their inhibition increases cardiomyocyte proliferation in the adult heart and improves left ventricular function after MI [115]. Moreover, miR-128 is upregulated during postnatal maturation and enforces cell cycle arrest; its deletion extends the proliferative window, promotes adult cardiomyocyte cell cycle re-entry, and attenuates fibrosis after MI, in part through modulation of the chromatin modifier suppressor of zeste 12 homolog, which suppresses p27 expression and activates positive cell cycle regulators such as cyclin E and CDK2 [116]. Collectively, miRNAs regulate cardiomyocyte proliferative capacity by coordinating cell cycle regulators, cytoskeletal remodeling, and developmental signaling pathways.

2. Long non-coding RNAs (lncRNAs), defined as nonprotein-coding transcripts longer than 200 nucleotides, have emerged as important regulators of cardiac development and aging [117]. Many lncRNAs function as competing endogenous RNAs (ceRNAs) that can “sponge” miRNAs, thereby diminishing the regulatory effects of miRNAs on their target mRNAs. The fetal lncRNA, named endogenous cardiac regeneration-associated regulator (ECRAR), promotes cardiomyocyte proliferation by forming a positive feedback loop with the E2F1-ERK1/2 pathway, thereby stimulating cell cycle progression [118]. In addition, Sirt1 antisense lncRNA enhances Sirt1 stability and contributes to cardiac regeneration [119]. Similarly, Snhg1 sustains PI3K/Akt signaling through a positive feedback loop with c-Myc, promoting cardiomyocyte proliferation and improving cardiac function after MI [120]. Conversely, several lncRNAs act as negative regulators of cardiac regeneration. CPR represses minichromosome maintenance complex component 3, a factor required for DNA replication [121]; CAREL functions as a ceRNA for miR-296, which targets Trp53inp1 and Itm2a, thereby modulating regenerative capacity [122]. LncDACH1 inhibits proliferation by enhancing YAP1 phosphorylation through interaction with protein phosphatase 1 catalytic subunit alpha, limiting YAP nuclear translocation [123]. Furthermore, CRRL inhibits cardiomyocyte regeneration by sponging miR-199a-3p and reducing homeodomain-only protein homeobox activity, thereby impairing neonatal and post-injury cardiac repair [124].
3. Circular RNAs (circRNAs) are covalently closed non-coding RNAs that exhibit high stability due to resistance to RNA exonuclease degradation [125]. Emerging evidence indicates that circRNAs modulate cardiomyocyte proliferation through regulation of transcriptional networks, signaling pathways, and miRNA activity. CircNfix functions as an inhibitor of cardiomyocyte proliferation by facilitating ubiquitin-mediated degradation of Y-box binding protein 1 (YBX1). Specifically, circNfix interacts with the E3 ubiquitin ligase NEDD4L to promote YBX1 ubiquitination, which in turn reduces cyclin A2 and cyclin B1 expression. CircNfix also serves as a sponge for miR-214, thereby enhancing Gsk3β expression and suppressing β-catenin activity [126]. Similarly, circNCX1 has been reported to suppress cardiomyocyte proliferation, as forced expression of circNCX1 inhibits proliferation whereas silencing endogenous circNCX1 exerts the opposite effect in vitro [127]. Inhibition of circALPK2 enhances the proliferation and therapeutic potential of hiPSC-CMs in MI models [128]. In contrast, circHipk3 promotes cardiomyocyte proliferation by increasing the acetylation and stability of the Notch1 intracellular domain. Additionally, circHipk3 sponges miR-133a to increase connective tissue growth factor expression, supporting angiogenesis and endothelial activation during cardiac repair [129]. CircRNAs also mediate intracellular communication. Hypoxia-induced CircWhsc1 is produced in endothelial cell-derived extracellular vesicles and transported to cardiomyocytes, where it activates the tripartite motif containing the 59/STAT3/cyclin B2 pathway to promote cardiac regeneration following MI [130].

Beyond rodent models, recent studies in large mammals have begun to highlight the functional relevance of circRNAs in cardiomyocyte cell cycle regulation and cardiac regeneration [131,132]. In the pig heart, transcriptomic profiling has revealed dynamic changes in circRNAs expression during postnatal maturation and following myocardial injury, with several circRNAs correlating with cardiomyocyte proliferative capacity and cell cycle-associated gene networks. Functional analyses further suggest that specific pig circRNAs participate in the regulation of cell cycle progression, cytoskeletal organization, and metabolic remodeling, underscoring their potential contribution to regenerative responses in a clinically relevant large animal model. Notably, Tang et al. identified the most pronounced differences in cell cycle-related pathways between postnatal day 1 and postnatal days 3, 7, and 28, uncovering ten previously uncharacterized circRNAs associated with cardiomyocyte cell cycle regulation in the pig heart [131]. Collectively, these findings support the emerging concept that circRNA-mediated regulation of cardiomyocyte proliferation may be leveraged for translational cardiac regeneration strategies. Beyond circRNAs, piwi-interacting RNAs (piRNAs) are an emerging class of non-coding RNAs with regulatory roles in cardiac repair. Notably, a myocardial cell proliferation–promoting piRNA (MCPPIR) enhances cardiomyocyte proliferation and cardiac repair by inducing o8G oxidation of POC1B mRNA [133].

## 5. Hormones and Growth Factors

Hormonal and growth factor signaling critically influence cardiomyocyte cell cycle regulation and regenerative capacity (Table S5). Thyroid hormone signaling has emerged as a key regulator of postnatal cell cycle exit [134]. In adult mouse hearts, inactivation of thyroid hormone signaling reduces cardiomyocyte polyploidization, delays cell cycle withdrawal and preserves regenerative potential by the downregulation of genes associated with oxidative phosphorylation, cardiac muscle contraction, and mitochondrial genes. Consistently, administration of thyroid hormone inhibits heart regeneration in zebrafish, implying that rising thyroid hormone levels contribute to the transition from a regenerative to a non-regenerative state in the heart. β-adrenergic signaling (β1-AR and β2-AR) inhibits neonatal heart regeneration after apical resection by reducing YAP m6A modification and YAP expression via subtype-specific regulation of methyltransferase-like 14 (β1-AR) and insulin-like growth factor 2 mRNA-binding protein 1 (β2-AR), with β2-AR showing a stronger inhibitory effect [135]. In addition, multiple growth factors promote cardiomyocyte cell cycle and cellular proliferation. Myeloid-derived growth factor (MYDGF), secreted predominantly by endothelial cells, activates the c-Myc/FOXM1 pathway and enhances cardiomyocyte proliferation and regeneration in both neonatal and adult mice following cardiac injury [136]. Fibroblast growth factor 10 similarly stimulates cardiomyocyte renewal while limiting fibrosis through modulation of MEIS1, Hippo signaling, and metabolic pathways [137].

Neuregulin1 (NRG1) signaling via its receptor Erb-B2 receptor tyrosine kinase 4 (ERBB4) has been studied, with genetic and pharmacological activation promoting cardiomyocyte proliferation and heart repair following injury [138]. However, conflicting findings indicate that recombinant NRG1 can preferentially stimulate DNA synthesis in non-cardiomyocytes and suppress baseline cardiomyocyte DNA synthesis in adult hearts [139]. A later study found that NRG1-induced CM proliferation diminished one week after birth owing to a reduction in ERBB2 expression. Activation of ERBB2, a co-receptor for NRG1, and its downstream pathways, including extracellular signal-regulated kinase, AKT, and GSK3β/β-catenin signaling, mediates cardiomyocyte dedifferentiation and proliferation, ultimately facilitating cardiac regeneration in neonatal, juvenile, and adult cardiomyocytes [140]. ERBB2 also promotes YAP activation and endothelial–mesenchymal transition-like processes during cardiac regeneration, leading to cytoskeletal remodeling, dissolution of cell junctions, migration, and extracellular matrix (ECM) turnover [141]. Recent work also implicates bone morphogenic protein 7 (BMP7) as a conserved pro-regenerative signal: loss-of-function reduces cardiomyocyte proliferation in neonatal mice and regenerating zebrafish, while overexpression or BMP7 treatment increases cardiomyocyte cycling via the bone morphogenetic protein receptor/activin receptor–SMAD5–ERK/AKT signaling axis [142]. Additional complexity arises from TGF-β family ligands, such as Mstnb and Inhbaa, which exert opposing effects after cardiac injury; notably, overexpression of *inhbaa* alone is sufficient to induce cardiomyocyte proliferation independently of the NRG–ERBB signaling pathway, whereas *mstnb* gain of function results in unresolved scarring following cardiac injury [143].

## 6. Contribution of Nonmyocytes to Heart Regeneration

Nonmyocyte populations, including immune cells, endothelial cells and fibroblasts, constitute the majority of cells in the heart and play distinct roles in maintaining cardiac structure and function under normal physiological conditions [144]. Prolonged ischemia leads to irreversible cardiomyocytes and endothelial cell death, initiating an inflammatory response that progresses through overlapping inflammatory, proliferative, and maturation phases [145]. Each phase is characterized by distinct cellular activities and interactions that collectively contribute to tissue repair and remodeling [146].

### 6.1. The Innate Immune Cells

The innate immune compartment of the heart comprises diverse myeloid populations, including monocytes, macrophages, dendritic cells, natural killer cells, and granulocytes [147]. Following MI, these cells orchestrate a dynamic immune response that critically shapes repair outcomes [144]. Single-cell RNA sequencing of neonatal hearts reveals a rapid increase in immune cell populations after injury, with distinct recruitment dynamics between regenerative (P1) and non-regenerative (P8) stages [148]. Regenerative P1 hearts display earlier and more coordinated macrophage and monocyte responses at one day post-infarction, whereas delayed immune recruitment in non-regenerative P8 hearts correlates with impaired repair. Administration of anti-inflammatory cytokine interleukin 13 (IL-13) promotes DNA synthesis and cell division in cardiomyocytes while reducing fibrosis in non-regenerative P6 mice. Deletion of IL-13 receptor subunit IL4Rα results in less cardiomyocyte proliferation during early postnatal development and noticeably hinders heart regeneration after injury in newborn mice [149]. These findings reflect that the timing and composition of innate immune activation contribute to regenerative ability.

Neutrophils are among the earliest immune responders after I/R injury, infiltrating the myocardium within hours and peaking during the first few days after injury [150,151,152,153]. Experimental modulation of neutrophil recruitment has yielded context-dependent results. While early inhibition of selectin-mediated adhesion can reduce acute tissue injury (30 min of coronary occlusion and 2 h of reperfusion), prolonged neutrophil depletion (a 60 min–24 h ischemic period) does not consistently improve infarct size and may worsen long-term cardiac function [154,155,156,157,158]. Neutrophil-derived factors, including neutrophil gelatinase-associated lipocalin, promote reparative macrophage polarization towards the M2c subtype and clearance of apoptotic cells, thereby facilitating resolution of inflammation and tissue remodeling [158]. These findings underscore the dual and temporally regulated roles of neutrophils in cardiac repair.

After the early neutrophil response, monocytes are recruited and mature into macrophages that dominate later inflammatory-to-reparative stages. These macrophages transition from pro-inflammatory to reparative states that promote angiogenesis, ECM remodeling, and scar maturation [152]. In regenerative species such as zebrafish and salamanders, macrophages are indispensable for heart regeneration. Their depletion disrupts vascularization, ECM remodeling, and cardiomyocyte survival, ultimately impairing functional recovery [159]. Notably, timely macrophage recruitment is critical; delayed recruitment results in prolonged expression of inflammation-related genes and defective neovascularization, reduced cardiomyocyte proliferation, and incomplete scar resolution [160,161]. Further, zebrafish lacking resident macrophages exhibit defects in heart regeneration. Importantly, these deficiencies cannot be compensated by circulating or monocyte-derived macrophages [161]. Consistent with macrophage-derived paracrine regulation of regeneration, conditioned medium from human iPSC-derived primitive macrophages promotes adult cardiomyocyte proliferation and cardiac regeneration [162], whereas M1 macrophage-derived exosomes can inhibit cardiomyocyte proliferation by delivering miR-155 and suppressing IL-6R/JAK/STAT3 signaling [163].

### 6.2. The Adaptive Immune Cells

The adaptive immune response after MI primarily involves T and B lymphocytes, which accumulate within the injured myocardium during the first week after injury [152,164]. These cells modulate inflammation, influence macrophage polarization, and shape myocardial remodeling, thereby affecting regenerative outcomes. T lymphocytes comprise CD4^+^ FOXP3^−^ T-helper (Th)/conventional CD4^+^ T cells, CD8^+^ T-cytotoxic (Tc) cells, and CD4^+^ FOXP3^+^ T-regulatory (Treg) cells, each exerting distinct effects following cardiac injury [151]. Both Tc and Th lymphocytes accumulate early after infarction and contribute to cardiomyocyte apoptosis and inflammatory amplification. Importantly, accumulating evidence supports subset- and time-dependent roles for CD4^+^ T cells after MI. In adult mice, CD4^+^ T cell deficiency is associated with increased left ventricular dilation and disturbed scar formation [165]. In contrast, CD4^+^ T cell ablation with GK1.5 in P8 mice results in mitigated cardiac fibrosis and increases cardiomyocyte proliferation [166]. In an I/R model, administration of the A2AR agonist ATL146e 5 min before reperfusion reduces myocardial T cell accumulation and infarct size [151]. CD8^+^ cytotoxic T cells also contribute to post-ischemic injury: CD8^+^ T cell depletion decreases cardiomyocyte apoptosis in the ischemic myocardium, blunts inflammatory responses, limits myocardial injury, and improves cardiac function [167].

Treg cells play a predominantly protective and pro-regenerative role. Treg numbers increase after MI and modulate macrophage polarization toward reparative phenotypes through the secretion of anti-inflammatory cytokines such as TGF-β1, IL-13, and IL-10 [168,169,170]. Ablation of Treg cells exacerbates cardiac injury, resulting in increased infarct size, worsened cardiac function, and elevated numbers of inflammatory myeloid cells and lymphocytes, whereas Treg expansion improves functional recovery and reduces fibrosis and cardiomyocyte apoptosis [168,171]. Importantly, in neonatal mice, Treg cells are essential for endogenous heart regeneration. Ablation of Tregs impairs regeneration, while adoptive transfer of Tregs promotes heart regeneration via paracrine mechanisms [172]. Several Treg-derived factors, such as C-C motif chemokine ligand 24, growth arrest-specific gene-6, amphiregulin, cystatin F, tumor necrosis factor superfamily member 11, interleukin 33, fibrinogen-like 2, matrilin 2, and insulin-like growth factor 2, have been shown to directly stimulate cardiomyocyte proliferation, underscoring their active role in regenerative signaling rather than solely immunosuppression [170,172,173].

Other T cell subsets contribute context-dependent effects. γδ T lymphocytes, a major source of IL-17A after I/R injury, promote inflammation and cardiomyocyte apoptosis; neutralization of IL-17A reduces infarct size, lowers cardiac troponin T levels, and improves cardiac function [174]. B lymphocytes also participate in post-infarction remodeling. B lymphocytes infiltration occurs early after injury, and B cell depletion through Baff receptor deficiency, antibody-mediated strategies using CD20- or Baff-specific antibodies, and B cell–selective *Ccl7* deficiency reduces inflammation and improves cardiac function [152,175]. Conversely, adoptive transfer of regulatory B cells ameliorates ventricular remodeling by reducing the expression of C-C motif chemokine receptor 2 on monocytes [176]. These findings suggest that, similar to T cells, B cell subsets exert both detrimental and protective effects depending on context and phenotype.

### 6.3. Cardiac Fibroblasts

Cardiac repair after MI relies on the mobilization and activation of fibroblasts and myofibroblasts which deposit ECM to provide structural support and reduce the risk of ventricular rupture. In regenerative species such as zebrafish, fibrosis is transient and tightly coordinated with cardiomyocyte proliferation [177]. Early stages following injury are marked by thrombosis, accumulation of fibroblasts, and collagen deposition. Subsequently, cardiac cells re-enter the cell cycle, migrate into the infarcted region, and regenerate new myocardium, resulting in scarless recovery [177]. Single-cell analyses have identified three distinct clusters of fibroblasts in zebrafish, *col11a1a*, *col12a1a*, and *nppc*. These cell populations are present at the peak regenerative stages and are required for effective repair; the depletion of specific pro-regenerative fibroblast population impairs cardiomyocyte proliferation and regeneration [178,179]. Specific ECM proteins, such as periostin (POSTN) and agrin, have been shown to stimulate cardiomyocyte proliferation and cardiac regeneration after MI in neonatal and adult hearts including large animal models [180,181,182,183].

In contrast, in adult mammals, persistent early fibrosis leads to adverse remodeling and functional decline. Genetic ablation of *Postn*-positive myofibroblasts or disruption of fibroblast-to-myofibroblast transition through muscleblind-like splicing regulator 1 compromise scar formation and lead to ventricular rupture, highlighting the essential structural role of myofibroblasts in acute repair [184,185,186,187]. Recombinant POSTN delivery has been proposed to promote cardiomyocyte proliferation and improve repair through the PI3K/GSK3β/cyclin D1 signaling pathway [180,181]. However, genetic manipulation of *Postn* expression in the heart does not affect myocyte content, cell cycle activity, or cardiac repair in mice [188]. In vitro experiments have shown that adult cardiac fibroblasts suppress the proliferation of immature cardiomyocytes, indicating context-dependent fibroblast-cardiomyocyte crosstalk [189]. While embryonic cardiac fibroblasts secrete ECM components, including fibronectin, collagen, and heparin-binding EGF-like growth factor, that promote cardiomyocyte proliferation via β1 integrin signaling, adult cardiac fibroblasts predominantly induce cardiomyocyte hypertrophy rather than proliferation [190]. In addition, neonatal fibroblast-derived slit guidance ligand 2 and nephronectin primarily facilitate the completion of cell division by supporting mitotic rounding and cytokinesis, without driving cardiomyocytes to re-enter the cell cycle or initiate DNA replication [191]. In contrast, versican acts earlier by activating integrin β1–ERK1/2–Akt signaling, thereby promoting cardiomyocyte cell cycle activity and improving repair from neonatal injury to adult MI [192]. Moreover, ECM-based therapies, including zebrafish-derived ECM, ECM microparticles, and platelet-derived gels, promote myocardial repair and functional recovery in adult mammalian hearts through mechanisms involving ERBB2 signaling and modulation of the injury microenvironment [193,194,195].

Taken together, fibroblasts serve a dual function in cardiac repair: they provide essential structural stabilization through ECM deposition while simultaneously influencing cardiomyocyte proliferation and remodeling. Importantly, the ECM is not merely a passive scaffold, but an active regulator of regeneration that modulates mechanical support, growth factor signaling, and cell–cell communication within the injured heart. The balance between transient reparative fibrosis and persistent pathological scarring appears to be a key determinant of regenerative success.

### 6.4. Endothelial Cells

In regenerative species such as zebrafish, rapid angiogenic sprouting into the injured region begins shortly after cardiac injury [196]. Regenerating coronary endothelial cells upregulate vascular endothelial growth factor C (VEGFc), which promotes endothelial proliferation and stimulates the expression of elastin microfibril interfacer 2b and chemokine C-X-C motif ligand 8a in both epicardium-derived cells and coronary endothelial cells. These molecular events collectively drive coronary revascularization during cardiac regeneration [196]. VEGFa-dependent endocardial guidance further supports the formation of a new coronary plexus that provides a structural and metabolic scaffold for cardiomyocyte repopulation of the injured myocardium [197]. Inhibition of VEGF signaling severely impairs revascularization and suppresses cardiomyocyte proliferation, resulting in persistent fibrosis. These findings suggest the requirement for timely endothelial invasion to enable effective regeneration [196]. In addition to VEGF signaling, endothelial-derived factors directly influence cardiomyocyte cell cycle activity. The endocardial gene, cellular communication network factor 2a, is induced following injury and regulates cardiomyocyte proliferation and scar resolution; its loss disrupts regeneration despite preserved vascular formation, while overexpression enhances cardiomyocyte proliferation and improves the resolution of transient collagen deposition in zebrafish, highlighting its importance in cardiac repair [198].

In mammalian models, angiogenesis occurs in the infarct border zone, with most new vessels arising from preexisting coronary vessel endothelial cells [199,200,201]. Cardiomyocytes contribute to this process by upregulating the transcription factor zinc finger E-box-binding homeobox 2, which induces secretion of paracrine factors such as thymosin β4 and prothymosin α to stimulate endothelial cell migration and angiogenesis [202]. In vitro studies have further demonstrated that endothelial networks improve cardiomyocyte survival and spatial organization, highlighting bidirectional crosstalk between these cell types [202]. In injured neonatal hearts, MYDGF is secreted predominantly by endothelial cells and promotes cardiomyocyte proliferation and regeneration [136]. Together, these studies underscore the critical role of endothelial cells in heart regeneration by providing both vascular support and pro-regenerative signals that promote cardiomyocyte survival, proliferation, and tissue repair.

## 7. Conclusions and Perspectives

Despite significant progress in elucidating the molecular mechanisms governing cardiomyocyte cell cycle regulation and cardiac regeneration, several fundamental challenges continue to limit both mechanistic insight and translational advancement. First, the extremely low cardiomyocyte cell cycle activity in adult mammalian hearts remains a major technical challenge. In most physiological and pathological settings, less than 1–2% of adult cardiomyocytes exhibit detectable cell cycle activity, complicating accurate quantification using conventional proliferation markers. Consequently, future studies will require highly sensitive and cardiomyocyte-specific approaches, including genetic lineage tracing, cell cycle phase reporters, and high-resolution or single cell-based analyses to reliably assess true cardiomyocyte proliferation in adult hearts. Second, the heavy reliance on rodent models highlights the need for expanded investigation in large animals. While rodent studies have been useful in identifying key regulatory pathways, species-specific differences in cardiomyocyte maturation, metabolism, and regenerative capacity may limit translational relevance. Large animal models such as pigs and non-human primates more closely mimic human cardiac structure and physiology, and therefore they are essential for validating regenerative mechanisms and assessing long-term functional integration and safety concerns. Third, despite the identification of multiple pro-cell cycle signaling pathways, clinically viable pharmacological strategies remain insufficient. Many experimental approaches have been relied on for genetic manipulation, which pose challenges for clinical translation. There is a pressing need to identify small molecules or drugs that can safely promote cardiomyocyte cell cycle re-entry while preserving contractile function and avoiding malignant transformation. Finally, precise cardiac-targeted delivery of pro-cell cycle therapeutics represents a critical translational hurdle. Systemic activation of proliferative pathways risks off-target effects in nonmyocytes populations, such as cardiac fibroblasts. The development of cardiomyocyte targeted delivery platforms, including biomaterial-based patches, nanoparticles, or ligand-directed carriers, will be essential to spatially and temporally restrict therapeutic activity to cardiomyocytes within the injured myocardium, thereby improving efficacy and safety. Taken together, overcoming these challenges will be essential for translating advances in cardiomyocyte cell cycle biology into safe and effective regenerative therapies for heart diseases.

## Data Availability

No new data were created or analyzed in this study.

## Supplementary Materials

The following supporting information can be downloaded at https://www.mdpi.com/article/10.3390/biology15070568/s1. Table S1. Cyclin/CDK pathway regulators of cardiomyocyte proliferation across species; Table S2. Transcription factors and co-activators that regulate cardiomyocyte proliferation across species; Table S3. Oxygen- and metabolism-related regulators of cardiomyocyte proliferation across species; Table S4. Epigenetic regulation of cardiomyocyte proliferation across species; Table S5. Hormonal and growth factor regulators of cardiomyocyte proliferation across species.

## Abbreviations

The following abbreviations are used in this manuscript:

GATA4	GATA binding protein 4
PGC-1α/β	Peroxisome proliferator-activated receptor gamma coactivator 1 alpha/beta
PPARα	Peroxisome proliferator-activated receptor alpha
NRF1/2	Nuclear respiratory factor 1/2
ERRα/β/γ	Estrogen-related receptor alpha/beta/gamma
CDKs	Cyclin-dependent kinases
CKIs	Cyclin-dependent kinase inhibitors
MKK3bE	A constitutively active mutant of MAP kinase kinase 3b
Mdm2	Murine double minute 2
TBX20	T-box transcription factor 20
YAP	Yes-associated protein 1
FOXK1	Forkhead box K1
FOXK2	Forkhead box K2
E2F4	E2F transcription factor 4
E2F2	E2F transcription factor 2
FOXM1	Forkhead box M1
Oct4	POU class 5 homeobox 1
Sox2	SRY-box transcription factor 2
Klf4	Krüppel-like factor 4
c-Myc	MYC proto-oncogene, bHLH transcription factor
MOB1B	MOB kinase activator 1B
HIF1α	Hypoxia-inducible factor 1 α
KLF1	Krüppel-like factor 1
FOXO3	Forkhead box O3
FOXP1	Forkhead box P1
MEIS1	Meis homeobox 1
Hand1	Heart and neural crest derivatives expressed 1
HOXB13	Homeobox B13
MEIS2	Meis homeobox 2
HIF2A	Hypoxia-inducible factor 2 alpha
HMGB2	High mobility group box 2
SPHK2	Sphingosine kinase 2
S1PR1	Sphingosine-1-phosphate receptor 1
Adssl1	Adenylosuccinate synthase-like 1
Phgdh3	Phosphoglycerate dehydrogenase
PSAT1	Phosphoserine aminotransferase 1
CTBP2	C-terminal binding protein 2
Sirt4	Sirtuin 4
ACSL1	Acyl-CoA synthetase long-chain family member 1
Cpt1b	Carnitine palmitoyltransferase 1B
HMGA1	High mobility group AT-hook 1
PTMA	Prothymosin alpha
HDAC7	Histone deacetylase 7
β1-AR	Beta-1 adrenergic receptor
β2-AR	Beta-2 adrenergic receptor
Mydgf	Myeloid-derived growth factor
FGF10	Fibroblast growth factor 10
NRG1	Neuregulin 1
ERBB4	Erb-b2 receptor tyrosine kinase 4
BMP7	Bone morphogenetic protein 7
mstnb	Myostatin b
inhbaa	Inhibin beta A subunit a
IL4Rα	Interleukin 4 receptor alpha
H3K27me3	Trimethylation of histone H3 lysine 27
STAT3	Signal transducer and activator of transcription 3
ECM	Extracellular matrix.
MI	Myocardial infarction
VEGFc	Vascular endothelial growth factor C
