# Supplementary material for: Molecular Regulation of Cardiomyocyte Cell Cycle and Regeneration
Source: Biology (Basel). 2026 Apr 2;15(7):568. doi: 10.3390/biology15070568 (PMC13072238; doi:10.3390/biology15070568)
Supplement: Supplementary file 1 [file biology-15-00568-s001.zip › biology-4202671-supplementary.pdf]

Table S1. Cyclin/CDK pathway regulators of cardiomyocyte proliferation across species				
	**Citation (PMID)**	**Mouse**	**Rat**	**hiPSC-CMs/hESC-CMs**
**Pro-proliferation**				
Cyclin A2[43]	15159393	Embryonic and neonatal: PCNA, PH3 Adult: CM number, PCNA, PH3		
Cyclin D1[44, 45]	9169494	Adult: DNA count, nucleation, thymidine incorporation		
	24821722	Adult: DNA count, nucleation, BrdU, Ki67 and PCNA		
cyclin D2[46, 47]	15576649	Adult MI: thymidine incorporation, CMs number, infarct size		
	29018036			Ki67, BrdU, PH3 and Aurora B
CDK1, CDK4, cyclin B1 and cyclin D1	29502971	Neonatal: EdU, PH3 Adult MI: EdU, PH3, lineage-tracing; heart function and scar size	Adult: EdU, PH3	EdU, PH3
**Inhibitor**				
p21, p27, p57[51]	21209082	NMCMs: BrdU, PH3 and Aurora B	Adult CMs: BrdU and Aurora B	
Cyclin G1[52]	20360255	P2, P6, P10, P14: DNA content, nucleation	NRCMs: thymidine incorporation, Aurora B, DNA content, nucleation	
p38[53]	15870258	Neonatal: BrdU, PH3 Adult: BrdU	Embryonic CMs: BrdU NRCMs: BrdU, PH3, Aurora B, survivin, CM number Adult: Ki67, BrdU, PH3 and Aurora B	
p53 and Mdm2[55]	28745540	Adult CMs: BrdU, PH3, survivin		

Abbreviations: CM, cardiomyocyte; adult CMs, isolated adult cardiomyocytes in vitro; NMCMs, isolated neonatal mouse cardiomyocytes in vitro; NRCMs, isolated neonatal rat cardiomyocytes in vitro; Adult, adult mouse in vivo experiments; Adult MI, adult mouse myocardial infarction model; Neonatal AR, neonatal mouse apical resection model;

Table S2. Transcription factors and co-activators that regulate cardiomyocyte proliferation across species				
	**Citation**	**Mouse**	**hiPSC-CMs/hESC-CMs**	**Rat**
**Pro-proliferation**				
GATA4[56]	18591257	Embryonic: Ki67, BrdU, PH3		

TBX20[57-60]	29903739	Embryonic: EdU		
	32850848			
	26841808	Adult: PH3, Aurora B, CM number<br>NMCMs: nucleation, PH3<br>Adult MI: PH3, Aurora B; heart function, infarct size		
	39028008			NRCMs: MTS
	23751911	Neonatal mice: PH3<br>Adult: PH3, nucleation		NRCMs: Ki67, BrdU, Aurora B, CM number
Notch[64-66]	18824567	NMCMs: BrdU, CM number		
	36583388		Ki67, pRB, cyclin D1	
	29186476	Neonatal sham/AR: BrdU, PH3		NRCMs: BrdU, PH3, Aurora B
FOXK1/FOXK2[61]	40128196	NMCMs: Ki67, PH3 and Aurora B<br>Neonatal: Ki67, PH3 and Aurora B, total CM number, nucleation<br>Neonatal MI: PH3, Aurora B; scar size and heart function<br>Adult MI model: Ki67, PH3 and Aurora B, nucleation, heart function, scar size		
E2F4[62]	19955219			NRCMs: BrdU and PH3
E2F4/E2F2[63]	15718499			NRCMs: BrdU, cell cycle by flow, nucleation,<br>flowcytometric BrdU-Hoechst assay
FOXM1[68]	36846912			
	21779394	Embryonic: Ki67, PH3<br>Postnatal (P11): PH3		
	41662454	Adult MI: Ki67, PH3; heart function, infarct size	Ki67, PH3, Aurora B, CM number	
Oct4, Sox2, Klf4, and c-Myc[72, 73]	34554778	Adult: EdU, PH3, Aurora B, Time-lapse imaging<br>Adult MI: EdU; heart function, scar size		

KLF1[74]	40145861	P7 NMCMs: Ki67, EdU, PH3<br>Neonatal: no difference in Ki67, PH3 and Aurora B<br>Neonatal MI: Ki67, PH3, Aurora B; heart function, scar areas<br>Adult MI: ki67, PH3, EdU, nucleation; Heart function		
Wnt/β-catenin[75-77]	30526659[75]	NMCMs: BrdU	Ki67, PH3, CM number, Survivin	
	32973339[76]	Neonatal/adult: PH3, EdU, CM number, nucleation and ploidy<br>Adult MI: EdU, PH3, Aurora B, nucleation, ploidy; heart function, fibrosis area		NRCMs: EdU, PH3, Aurora B, CM number,
	39837836[77]	NMCMs: EdU<br>Neonatal AR: EdU, PH3; fibrotic area<br>Adult MI: EdU, lineage-tracing; heart function, fibrotic area	EdU	
Hippo[78]	24255096[78]	Adult: EdU, ki67, Aurora B, nucleation, ploidy<br>P8 AR: lineage tracing; heart function, scar size<br>P8 and Adult MI: EdU, Aurora B, nucleation, ploidy; heart function, infarct size		
YAP[79-82]	40555744[79]	NMCMs: EdU, Ki67, PH3<br>Neonatal sham/AR: Ki67, PH3; heart function,<br>Adult: PH3, Ki67<br>Adult MI: Ki67, PH3; heart function, fibrotic area<br>Fetal and postnatal mice: EdU, PH3		NRCMs: BrdU, PH3, Aurora B
	22308401[80]	Fetal: EdU, PH3, CM number<br>P15 heart: EdU, PH3		NRCMs: BrdU, PH3, Aurora B
	23918388[81]	Neonatal mice: PH3<br>P7 and Adult MI: PH3, fibrotic area, heart function<br>Adult: PH3<br>Neonatal MI: fibrotic area		
	39392007[82]	Adult: EdU, PCNA, labeling CM clone, nucleation		
**Inhibitor**				
FOXO3[70]	40087279	Neonatal: EdU, PH3, PCNA, Auora B, CM number, nucleation, Heart function<br>Neonatal P1 AR: Ki67, PH3 and heart function<br>Neonates P7 MI: Ki67, PH3, Aurora B and heart function, scar size		
FOXP1[71]	39899693	Neonatal AR: Ki67, Aurora B<br>Adult: EdU, ki67, PH3, Aurora B; heart function	EdU, PH3	
MEIS1[84]	23594737	P14 mice: PH3, BrdU, Aurora B.<br>Neonatal: PH3<br>Neonatal MI: heart function<br>Adult: total CM number, nucleation		NRCMs: PH3

HOXB13[85]	32499640	P14: PH3<br>Adult: PH3 and Aurora B, lineage tracing<br>Adult MI: BrdU PH3, heart function, no difference in scar formation		
MEIS2[86]	31315484		EdU, Ki67, PH3and aurora B	NRCMs: EdU, Ki67, Ph3 and aurora B<br>Adult MI: Ki67, Aurora B, infarct size, heart function
**Abbreviations**: CM, cardiomyocyte; adult CMs, isolated adult cardiomyocytes in vitro; NMCMs, isolated neonatal mouse cardiomyocytes in vitro; NRCMs, isolated neonatal rat cardiomyocytes in vitro; Adult, adult mouse in vivo experiments; Adult MI, adult mouse myocardial infarction model; Neonatal AR, neonatal mouse apical resection model.				

**Table S3. Oxygen- and metabolism-related regulators of cardiomyocyte proliferation across species**			
	**Citation**	**Mouse**	**Rat**
**Pro-proliferation**			
Hypoxemia[89]	27798600	Adult: CM number, BrdU, PH3, Aurora B<br>Adult MI: BrdU, PH3 and Aurora B, nucleation; heart function, fibrosis	
HIF2A[90]	38455514	Adult: CM number<br>Adult MI: PH3, linear tracing, heart function, fibrosis	
HMGB2[91]	41092376	NMCMs: Ki67, EdU, PH3, Aurora B<br>Neonatal AR: Ki67, Edu, PH3 and Aurora B; heart function, fibrosis.<br>Adult MI: Ki67, Edu, PH3 and Aurora B, nucleation, ploidy, CM number; heart function, scar area	
Glycolysis[92]	32648304		NRCMs: Ki67
Malonate[92]	33666092	P1 MI: PH3, heart function, fibrotic scar<br>P7 MI: PH3, Aurora B; fibrotic scar and heart function<br>Adult MI: BrdU, PH3, Aurora B; heart function, infarct size	
SPHK2[96]	38367623	Neonatal AR: PH3, Aurora B; heart function, scar size<br>P8 AR: FUCCI, PH3, Aurora B; heart function, scar size<br>Adult MI: Ki67, PH3, Aurora B, Lineage-tracing; heart function, scar size	NRCMs: PH3, Aurora B
S1PR1[97]	39816679	Neonatal AR: Ki67, PH3, EdU, Aurora B; heart function, scar size<br>Adult MI: Ki67, PH3, EdU, Aurora B, heart function, scar size	
Adssl1[98]	39471248	Neonatal AR: Ki67, PH3, Aurora B, heart function<br>Neonatal: P7 and P14 Ki67, PH3; no difference at P28<br>Adult MI: Ki67, PH3, Aurora B; heart function, scar size	NRCMs: Ki67, PH3
Xanthine oxidase[99]	39637598	Primary neonatal CM: EdU<br>P7 and P14: (PH3).<br>P7 MI: PH3, heart function, scar size	
Phgdh3[100]	40930719	P7 heart: Ki67, PH3<br>Neonatal AR: Ki67, PH3; heart function<br>Adult MI: Ki67 and PH3; heart function, scar size	NRCMs: ki67, PH3
PSAT1[101]	40756345	Adult MI: BrdU, PH3, Lineage-tracing, cm NUMBER, nucleation, heart function, scar size,	NRCMs: Ki67, PH3, CM number
CTBP2[102]	40140769	NMCMs: Ki67, EdU, CM number<br>Adult MI: BrdU, Ki67, PH3, heart function, scar size	
**Inhibitor**			

Sirt4[88]	40842073	NMCMs: PH3, Ki67 Aurora B.<br>Neonatal AR: PH3, Ki67 Aurora B, nucleation; heart function, scar size<br>P7 MI: PH3, Ki67, Aurora B; heart function, scar size<br>Adult I/R: PH3, Ki67, Aurora B; heart function, scar size	
ACSL1[94]	35122795	Adult heart: EdU, Ki67, PH3, Aurora B<br>Primary neonatal CMs: EdU, Ki67, PH3, Aurora B, flow<br>Adult MI: EdU, heart function	
Cpt1b[95]	37758950	NMCMs: EdU, Ki67, PH3<br>Adult: EdU, ki67, PH3 and Aurora B, CM number<br>Adult I/R: EdU, ki67, PH3 and Aurora B; infarct area	
**Abbreviations**: CM, cardiomyocyte; adult CMs, isolated adult cardiomyocytes in vitro; NMCMs, isolated neonatal mouse cardiomyocytes in vitro; NRCMs, isolated neonatal rat cardiomyocytes in vitro; Adult, adult mouse in vivo experiments; Adult MI, adult mouse myocardial infarction model; Neonatal AR, neonatal mouse apical resection model.			

**Table S4. Epigenetic regulation of cardiomyocyte proliferation across species**				
	**Citation**	**Mouse**	**hiPSC-CMs/hESC-CMs**	**Rat**
**Chromatin-based epigenetic regulation**				
**Pro-proliferation**				
α-ketoglutarate[95]	37758950	NMCMs: Ki67, EdU, PH3		
HMGA1[103]	39747457	Adult MI: Ki67, EdU, Aurora B, Heart function, scar size		NRCMs: Ki67, EdU<br>Neonates: EdU, Ki67, PCM
PTMA[104]	40408476	Embryonic and neonatal CMs: FUCCI, EdU, PH3, and Aurora B<br>Neonatal and adult: EdU, PH3 and Aurora B.<br>P1 AR: EdU, PH3, and Aurora B; heart function<br>P8 MI: EdU, Ph3, Aurora B; heart function, scar size<br>Adult MI: EdU, PH3, Auorora B, isolated CM number, single color cells cluster; heart function, scar size	EdU, PH3 and Aurora B	
HDAC7[105]	39394661	NMCMs: Ki67, PH3		
**Non-coding RNAs**				
**miRNA**				
**Pro-proliferation**				
miR-17/92[107]	23575307	Embryonic and postnatal hearts: PH3<br>P15 heart: EdU, PH3<br>Adult mice: CM number, nucleation<br>Adult MI: EdU, PH3, CM number; heart function, scar size		NRCMs: EdU, Auora B
miR-199a-3p/miR-590-3p[106, 113]	23222520[105]	NMCMs: EdU, PH3 and Aurora B.<br>Neonatal mice: EdU, PH3<br>Adult MI: EdU, heart function, infarct size		NRCMs: Ki67EdU, PH3 and Aurora B.<br>Adult CMs: EdU and CM number<br>Neonatal: EdU, CM number
	28077443[112]	Adult MI: EdU, PH3, Aurora B; heart function, infarct size		

miR-302/367 clusters[108]	25787764	E14.5, Ki67<br>embryonic and postnatal hearts (PH3, E18.5 and P20).<br>Primary neonatal CM: Ki67.<br>Adult: BrdU, PH3, Aurora B, nucleation, CM number<br>Adult MI: prolonged miR302-367 overexpression increases PH3 CMs, reduces fibrotic scar size but compromises cardiac function after MI.Transient miR-302 mimic therapy promotes cardiac regeneration and improves function of injured hearts.		
miR-431[109]	38282147	NMCMs: EdU, PH3, Auora B<br>Adult: EdU<br>Adult MI: EdU, heart function		
miR-130b-5p[110]	37933895	Adult MI: Ki67 and PH3; heart function, scar size	mESC-CMs: Ki67, PH3, Aurora B	
miR-1825[111]	28670398	P12 mice: EdU<br>Adult MI: EdU, heart function		NRCMs: EdU, Ph3, Aurora B, CM number, nucleation
miR-19a/19b[112]	30996254	Adult MI: EdU, PH3 and Aurora B, CM number; heart function, infarct size		
miR-1248[113]	28077443	NMCMs: EdU		NRCMs: EdU
miR-33b[113]	28077443	NMCMs: EdU, PH3, no difference of Aurora B, but CM number still increased		NRCMs: EdU, PH3 and Aurora B
**Inhibitor**				
miR-1[114]	31700891	NMCMs: cell cycle by Flow, CM number, BrdU		
miR-15 family,<br>such as miR-195[115]	23248315	Neonatal MI: PH3, heart function<br>Adult MI: PH3, heart function		
miR-128[116]	29453456	Neonatal: Ki67, EdU, heart function<br>Neonatal AR: EdU, heart function<br>Adult: Edu, CM number, nucleation<br>Adult MI: EdU, Aurora B, heart function, scar size		NRCMs: EdU, PH3 and Aurora B
**Long non-coding RNAs (lncRNAs)**				
**Pro-proliferation**				
ECRAR[118]	30528086			NRCMs: EdU, PH3, Aurora B, CM number, time-lapse video of cell division<br>P21 heart: Ki67, EdU, PH3 and Aurora B, CM number<br>P1 heart: EdU, PH3, fibrosis area<br>P7 MI: P14, EdU, PH3, Aurora B<br>Adult MI: EdU, PH3, heart function, infarction size
Sirt1 Antisense Long Noncoding RNA[119]	30608184	NMCMs: Ki67, PH3, CM number<br>Neonatal: Ki67, Ph3<br>Adult: Ki67, PH3<br>Adult MI: Ki67, PH3; heart function, infarct size		
Snhg1[120]	34646377	P7 neonatal CMs and mice: in vitro and in vivo, EdU, PH3 and Aurora B, cell cycle by flow, nucleation<br>Neonatal MI: heart function, scar size<br>Adult: EdU, Ki67, PH3 and Aurora B , CM number, nucleation<br>Adult MI: Ki67, PH3, heart function, scar size		
**Inhibitor**				

Long Noncoding RNA CPR [121]	30832495	NMCMs: PH3, EdU, Aurora B, CM number<br>P14 mice: PH3, EdU and Aurora B<br>Adult mice: PH3, EdU and Aurora B, nucleation, CM number<br>Neonatal MI: EdU and PH3; fibrosis area<br>Adult MI: EdU; heart function, scar size		
CAREL[122]	30056829	NMCMs (P7): EdU, PH3, Aurora B, Neonatal: PH3, Aurora B, nucleation<br>Neonatal AR: PH3, Aurora B; heart function<br>Neonatal P7 MI: PH3, Aurora B; heart function, infarct size	PH3, Aurora B	
LncDACH1[123]	31969690	NMCMs: PH3<br>Neonatal mice: PH3 and Aurora B<br>Neonatal mice AR: EdU, PH3 and Aurora B, heart function<br>Adult mice: PH3, Aurora B<br>Adult MI: PH3, Aurora B; heart function, scar size	PH3, EdU	
CRRL[124]	30125571			Adult MI: heart function, infarct size<br>NRCMs: EdU, Ki-67, PH3, Aurora B<br>P1, P7 hearts: EdU, Ki-67, PH3, Aurora B<br>Neonatal MI: PH3, EdU, Fibrosis size
**Abbreviations**: CM, cardiomyocyte; adult CMs, isolated adult cardiomyocytes in vitro; NMCMs, isolated neonatal mouse cardiomyocytes in vitro; NRCMs, isolated neonatal rat cardiomyocytes in vitro; Adult, adult mouse in vivo experiments; Adult MI, adult mouse myocardial infarction model; Neonatal AR, neonatal mouse apical resection model.				

Table S5. Hormonal and growth factor regulators of cardiomyocyte proliferation across species

	Citation	Mouse	Rat
**Inhibitor**			
Thyroid[114]	30846611	Neonatal: EdU, KI67 and PH3<br>Adult MI: EdU, ki67, PH3, Aurora B, CM number; heart function, scar size	
β1-AR and β2-AR[135]	40229267	Neonatal AR: PH3, KI67, aurora B; heart function, regeneration number of mice	
**Pro-proliferation**			
MYDGF[136]	32802181	Neonatal AR: PH3, Ki67, Aurora B, nucleation; heart function<br>neonatal primary CMs: PH3, Ki67, Aurora B	
FGF10[137]	34755840	Adult MI: Ki67, PH3 and Aurora B; heart function, fibrosis.	
NRGA[138]	19632177	Neonatal: BrdU incorporation, CM number<br>Adult: BrdU, PH3 and Aurora B<br>Adult MI: BrdU, PH3, Aurora B; heart function, scar size	NRCMs: DNA synthesis, DAPI, PH3 and video microscopy.
	25545368	Adult: BrdU<br>Adult MI: tritiated thymidine	

	25848746	NMCMs: TMRE, Ki67, Ph3, Aurora B<br>Neonatal MI: Ki67, Aurora B, heart function, scar volume<br>Adult MI: Ki67, Aurora B, heart function, scar volume	
BMP7[142]	38678558	NMCMs: BrdU, Ki67, Aurora B and TMRE<br>Neonatal: Ki67, BrdU<br>Adult MI: Ki67 and Aurora B	
**Abbreviations**: CM, cardiomyocyte; adult CMs, isolated adult cardiomyocytes in vitro; NMCMs, isolated neonatal mouse cardiomyocytes in vitro; NRCMs, isolated neonatal rat cardiomyocytes in vitro; Adult, adult mouse in vivo experiments; Adult MI, adult mouse myocardial infarction model; Neonatal AR, neonatal mouse apical resection model.			
